# Semaphorin-1a-like gene plays an important role in the embryonic development of silkworm, *Bombyx mori*

**DOI:** 10.1371/journal.pone.0240193

**Published:** 2020-10-02

**Authors:** Anli Chen, Qiongyan Li, Pengfei Liao, Qiaoling Zhao, Shunming Tang, Pingyang Wang, Gang Meng, Zhanpeng Dong

**Affiliations:** 1 The Sericultural and Apicultural Research Institute, Yunnan Academy of Agricultural Sciences, Mengzi Yunnan, China; 2 The Key Sericultural Laboratory of Shaanxi, Ankang University, Ankang Shaanxi, China; 3 The Sericultural Research Institute, Jiangsu University of Science and Technology, Zhenjiang, Jiangsu, China; University of Bari, ITALY

## Abstract

Fuyin-lethal red egg (Fuyin-*lre*) is a red egg mutant discovered from the germplasm resource Fuyin of *Bombyx mori*. The embryo of Fuyin-*lre* stops developing at the late stage of gastrulation due to chromosome structural variation. In this work, precise mutation sites at both ends of the mutated region were determined, and two inserted sequences with lengths of 1232 bp and 1845 bp were obtained at both ends of the mutation region. Interestingly, a *bmmar1* transposon was detected in the inserted 1845 bp sequence. *Bmmar1* possesses features of the *Tcl/mariner* superfamily of transposable elements (TEs), which belongs to class II TEs that use a DNA-mediated “cut and paste” mechanism to transpose. This finding suggests that Fuyin-*lre* mutation might be related to the “cut and paste” action of *bmmar1*. The mutation resulted in the deletion of 9 genes in the mutation region, of which the red egg gene *re* (BMSK0002766) did not affect embryonic development of *B*. *mori*, and the BMSK0002765 gene was unexpressed during the early stage of embryonic development. The RNA interference results of the remaining 7 genes suggest that the semaphorin-1a-like gene (BMSK0002764) had a major contribution to the embryonic lethality of Fuyin-*lre*.

## 1. Introduction

The silkworm, *Bombyx mori* (*B*. *mori*), is an important economic and lepidopteran model insect with abundant genetic resources. This species is a desirable material for genetic and gene mutation research particularly because of the publication of its complete genome sequence [1–4] and a high-density linkage map [5,6]. Numerous genetic variations in the traits of this moth at every life stage have been discovered, and more than 1,000 *B*. *mori* genetic materials, including mutant genes, chromosomal variation strains, and genes with exceptional qualities, are currently maintained as genetic resources [7].

The egg stage is the first and the most important period in the silkworm life cycle. The quality of silkworm eggs directly affects the development of larvae, pupae, and moths. The egg stage is an important indicator to measure the superiority of silkworm varieties. Therefore, egg mutations, especially those that substantially affect egg development, must be studied. Lethal mutations are an important class of egg mutations, and they are mainly divided into two categories. The first category involves the incomplete egg shell structure caused by egg shape mutations, and the second category includes the abnormal embryonic development caused by the mutation of genes that play key roles in this development stage. The collapsing dead egg mutant *Gr*^*col*^ is caused by the production of an eggshell in which most proteins are underrepresented to varying degrees [8]. The eggs laid by homozygous recessive “Ming” lethal egg mutants (*l-e*^*m*^) lose water and become concaved around 1 h after egg laying, eventually forming a triangular shape on the egg surfaces [9,10]. These are lethal mutations caused by an incomplete egg structure, which is controlled by the recessive genetics and follows the pseudo maternal inheritance of eggshell traits. The two Z chromosomes of the male sex-linked balanced lethal silkworm strain contain non-allelic, tightly linked recessive embryonic lethal genes l1 and l2, whose lethal periods are the body pigmentation stage and the G2 period, respectively [11,12]. This kind of mutation belongs to the second category of egg lethal mutation.

Transposable elements (TEs) are one of the factors that cause the mutation of silkworm. TEs have the potential to alter the genome structure and play a major role in genome evolution [13]. Previous studies showed that the activity of retrotransposons was responsible for several types of mutations in the silkworm genome, including insertions and retrotransposon-associated genomic deletions [14–16]. *Bmmar1* is one of the TEs found in the silkworm genome, with approximately 2,400 copies [17]. It has features of the *Tcl/mariner* superfamily of TEs, which belong to the class II TEs that use a DNA-mediated “cut and paste” mechanism to transpose [18]. *Tcl/mariner* transposases contain the DNA binding domain and the catalytic domain. The catalytic domains of their transposases include a conserved motif DDD/E, which play important roles in the process of transposition [19]. The active *Tcl/mariner* transposons could be used as powerful molecular tools in the procedures of transgenesis and insertion mutagenesis [20,21].

**Fig 1:**
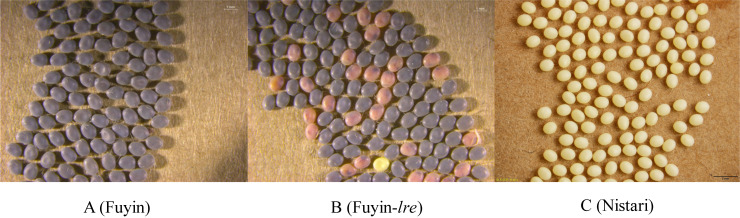
Egg phenotype of Fuyin, Fuyin-*lre*, and Nistari at 72 h after egg laying. (A) Wild-type silkworm variety Fuyin. The eggs are yellow when they are laid and then turn gray after 72 h due to diapause. (B) Fuyin-*lre*, red egg mutant of Fuyin. Some eggs turn gray and the other eggs turn red 72 h after egg laying. The gray eggs can hatch after incubation, but red eggs cannot hatch. (C) Diapause-free silkworm variety Nistari. The eggs are yellow during the whole egg period. The eggs laid by Fuyin are diapause eggs, and they need to be treated with hydrochloric acid to terminate the diapause. Silkworm eggs after injection are fragile, and hydrochloric acid treatment will cause the death of the eggs. The eggs laid by Nistari are diapause-free eggs and are widely used in microinjection experiments. Diapause-free eggs can develop normally without hydrochloric acid treatment. Therefore, the diapause-free eggs laid by Nistari were used in RNAi.

Fuyin-*lre* is a novel red egg mutant of silkworm and differs from previously reported red egg mutants because of its embryonic lethality (Fig 1B). Previous studies showed that the Fuyin-*lre* mutant is a product of the structural variation of chromosomes [22]. In the present work, we found that the occurrence of mutations may be related to the “cut and paste” function of the *bmmar1* transposon and proved that the semaphorin-1a (Sema-1a) gene has an important effect on the development of silkworm embryos. Semaphorin-1a belongs to the Sema protein family, which is a major group of axon guidance molecules [23,24]. During *Drosophila* embryonic neural development, Sema-1a functions not only as a ligand for PlexA but also as a receptor for unknown ligands [25]. In the peripheral nervous system (PNS), bidirectional signaling of Sema-1a plays an essential role in motor axon pathfinding by mediating axon–axon repulsion [26]. However, to our knowledge, no report exists about the impact of Sema-1a on embryonic development. This article preliminarily clarified the mutant form of Fuyin-*lre* and the role of Sema-1a on the embryonic development of silkworm.

## 2. Materials and methods

### 2.1 Silkworm strains

Wild-type Fuyin (Fig 1A) and its mutant Fuyin-*lre* with embryonic lethality (Fig 1B) were preserved by the Sericultural and Apicultural Research Institute, Yunnan Academy of Agricultural Sciences. Diapause-free silkworm variety Nistari (Fig 1C) was provided by the Sericultural Research Institute, Chinese Academy of Agricultural Sciences. All *B*. *mori* individuals were fed fresh mulberry leaves at a constant temperature of 25 ± 0.5°C and constant humidity of 75% to 80%.

### 2.2 Genomic DNA extraction and inverse Polymerase Chain Reaction (iPCR)

iPCR was used to determinate the mutation sites. Genomic DNA of Fuyin and Fuyin-*lre* individuals was extracted from the wild-type and mutant eggs in accordance with previously described methods [27]. The concentration and purity of genomic DNA were determined using an ultra-micro spectrophotometer (NanoPhotometer N60) at a 260/280 absorbance ratio, and the purified DNA was stored at −20°C.

For iPCR, 5 μg genomic DNA of the mutant strain Fuyin-*lre* was digested with restriction enzymes XhoⅠ, ApaLⅠ, and HindIII at 37°C for 30 min. After denaturation at 65°C for 15 min, the digested DNA was precipitated by ethanol and dissolved in 20 μL ddH_2_O. A total of 1 μL digested DNA was ligated by Solution I (TaKaRa), and the product was used as the template for iPCR. The iPCR products were sequenced by Sangon Biotech (Shanghai) Co., Ltd. The sequence obtained by sequencing was blasted on the silkworm genome to determine the position of the sequence on the chromosome. S1 Table lists the primer sequences of iPCR.

### 2.3 RNA preparation and quantitative Reverse Transcription PCR (qRT-PCR)

The eggs laid by Fuyin and Fuyin-*lre* were separately treated and incubated. Every 24 h after incubation, 0.5 g normal eggs laid by Fuyin and red eggs laid by Fuyin-*lre* were collected for total RNA extraction by using the RNAiso Plus (TaKaRa) kit. The extracted RNA was digested by DNase I (TaKaRa) to remove DNA residues. The concentration and purity of total RNA were determined using an NP60 Touch (IMPLEN). The qualified RNA was used for qRT-PCR.

A total of 500 ng of the previously mentioned total RNA was used to synthesize first-strand cDNA with a PrimeScript reverse transcriptase kit (TaKaRa) in a 10 μL reaction system. The cDNA products were diluted fivefold with ddH2O. qRT-PCR was performed in a StepOnePlus Real-Time PCR system (Applied Biosystems) using default parameters. Each 20 μL qRT-PCR reaction system consisted of 9 μL SYBR Premix Ex Tag (Roche, 2×), 0.8 μL specific primers, 1 μL cDNA template, and 9.2 μL ddH2O. Each PCR reaction was repeated thrice. The housekeeping *B*. *mori* actin 3 gene (A3, GenBank ID: NM_001126254) [28] was used as a reference to eliminate bias among samples, and qRT-PCR results were converted and calculated by using the 2^-ΔΔ*C*^_T_ method [29]. The qRT-PCR primer sequences are listed in S1 Table.

### 2.4 RNA interference (RNAi)

The corresponding small interfering RNAs (siRNAs) were designed and synthesized based on the coding sequences (CDS) of candidate genes. In addition, the ddH_2_O and enhanced green fluorescence protein (EGFP) sequence was synthesized by RiboBio Company (http://www.sirna.cn/index.aspx) as negative control. Three target regions were chosen per gene to synthesize siRNA, and each siRNA dry powder was separately diluted to 20 ng/μL by using RNase-free water. Subsequently, the three siRNAs corresponding to each gene were mixed in an equal volume of 1:1:1 and stored at −80°C. The siRNA sequences are listed in S2 Table.

RNAi was performed on a TransferMan 4r microinjection system (Eppendorf). Liquid glue was applied to sticky paper, and a steel ring was placed on the sticky paper after drying. After mating, the female moth of diapause-free silkworm variety Nistari was placed in the steel ring, and the steel ring was placed in a dark environment for the female moth to lay eggs. The silkworm eggs were eluted from the sticky paper after laying and washed thrice with sterile water. Subsequently, the silkworm eggs were placed neatly on a glass slide for microinjection. The microinjection was performed within 4 hours after egg laying. Each silkworm egg was injected with about 5 nL siRNA mixture. After injection, the silkworm eggs were incubated at 25°C and 75% relative humidity. Every 24 h after incubation, total RNA of 20 injected silkworm eggs was extracted for qRT-PCR. RNA extraction and qRT-PCR methods are the same as 2.3. RNAi had been successfully used by the authors of this paper to verify the function of *BmEP80* in silkworm [10]. The difference in the present study is that we reduced the amount of injected siRNAs because the eggs were smaller.

## 3. Results and discussion

### 3.1 Determination of mutation sites at both ends of the mutated region

Previous studies showed that a sequence of more than 280 kb on chromosome 5 was deleted and replaced by a new fragment of unknown size in the Fuyin-*lre* mutant [22]. The mutation sites at both ends of the mutation region were determined within the 234 bp and 265 bp regions, respectively (Fig 4 in reference 22).

**Fig 2:**
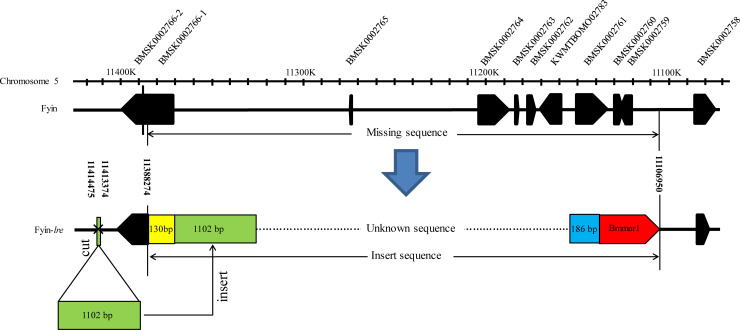
Structure diagram of the mutation region in Fuyin-*lre*. In the Fuyin-*lre* mutant, the 281325 bp fragment starts from 11106950 bp and ends at 11388274 bp on chromosome 5 is replaced by some new DNA fragments. The 1232 bp and 1845 bp sequences were obtained at both ends of the mutation region by iPCR. 1–130 bp of the 1232 bp sequence and 1–186 bp of the 1845 bp sequence have multiple copies in the silkworm genome. 131–1232 bp of the 1232 bp sequence has only one single copy on the silkworm genome, which is derived from 11413374 bp to 11414475 bp of chromosome 5. A *bmmar1* transposon was detected in 187–1845 bp of the 1845 bp sequence.

**Fig 3:**
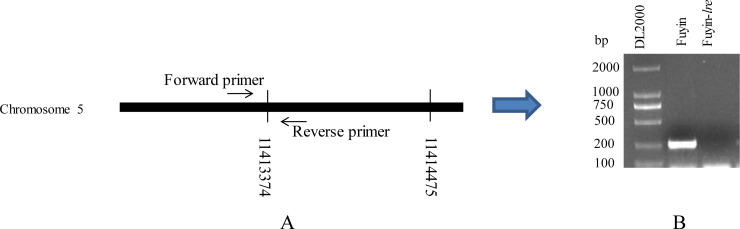
Identification of the insertion form of 131–1232 bp of the 1232 bp sequence. (A) The position of primers used for PCR amplification; (B) PCR amplification results. PCR amplification could be performed when the Fuyin genome is used as a template but not when the Fuyin-*lre* genome is used. This finding indicates that 131–1232 bp of the 1232 bp sequence was inserted into the mutated region after cutting.

In this study, the mutation sites at both ends of the mutation region were determined by iPCR. The mutation region starts from 11106950 bp and ends at 11388274 bp (281325 bp) on chromosome 5 (SilkDB 3.0). We found a 1232 bp and an 1845 bp sequence at either ends of the mutant region (Fig 2). Sequence alignment revealed that the 1232 bp sequence was composed of two sequences (Fig 2). The first sequence, which has a length of 130 bp, has multiple copies in the silkworm genome, and determining the origin of such sequence was difficult. The second sequence, which has a length of 1102 bp, has only a single copy on the silkworm genome, which is derived from 11413374 bp to 11414475 bp of chromosome 5. A pair of primers on both sides of 11413374 bp site (chromosome 5) was designed and PCR amplification was performed by using Fuyin and Fuyin-*lre* genomes as templates to determine whether the second sequence was inserted by a duplication or an excision event (Fig 3A). The results showed that PCR amplification could be performed when the Fuyin genome is used as a template, but it could not be performed when the Fuyin-*lre* genome is used as a template (Fig 3B). This finding indicates that the second sequence was inserted into the mutated region by an excision event. The primer sequences are listed in S1 Table.

Sequence alignment showed that the 1845 bp sequence at the other end was also composed of two sequences (Fig 2). The first sequence, which has a length of 186 bp, has multiple copies in the silkworm genome, and determining the origin of such sequence was difficult. Interestingly, a *bmmar1* transposon was detected in the second sequence (Fig 2). *Bmmar1* is derived from a transposase-mediated transposon, which includes approximately 2,400 copies in the silkworm genome [17]. *Bmmar1* has the features of the *Tcl/mariner* superfamily of TEs, which belong to the class II TEs that use the DNA-mediated “cut and paste” mechanism to transpose [18]. Considering that the 281325 bp sequence was deleted from chromosome 5 of Fuyin-*lre*, and the inserted length of 1102 bp was cut from 11413374 bp to 11414475 bp of chromosome 5, the Fuyin-*lre* mutation might be related to the “cut and paste” action of *bmmar1*.

We attempted to determine the complete sequence information of the mutation region through genome resequencing, large-fragment PCR, and other methods. However, we failed, probably because the sequence inserted in the mutation region is too large.

### 3.2. Effects of nine genes in the mutated region on embryonic development

Previous studies showed that nine genes, including the red egg gene *re* (BMSK0002766), were missing in the mutated region and had an almost undetectable expression [22]. Among these nine genes, *re* was partially lost, and the remaining eight genes disappeared completely [22]. The sequence analysis of *re* by the predecessors showed that the embryos of *re* mutant developed normally after the frameshift mutation [22] or structural variation [30] of *re*. The RNAi results of *re* showed that the defect of the *re* gene was responsible for the *re* phenotype and that it functioned in the ommochrome biosynthesis pathway of *B*. *mori* [30]. These results indicate that the *re* gene was responsible for the *re* phenotype and did not affect the embryonic development of *B*. *mori*. The results of DGE and qRT-PCR on BMSK0002765 (SilkDB 3.0, https://silkdb.bioinfotoolkits.net/) showed that the BMSK0002765 gene was unexpressed during the early stage of embryonic development [22]. Therefore, BMSK0002765 appeared to have no association with the embryonic death of Fuyin*-lre*. The remaining seven genes were expressed during the embryonic development of Fuyin. The Fuyin*-lre* embryos stop developing at 48 h after egg laying; therefore, the expressions of the remaining 7 genes at 24 hours and 48 hours after egg laying were analyzed. The qRT-PCR results showed that the seven genes were ranked as follows in descending order of expression: BMSK0002762, BMSK0002764, BMSK0002759, KWMTBOMO02783 (http://sgid.popgenetics.net/), BMSK0002761, BMSK0002763, and BMSK0002760 (S1 Fig). BMSK0002760 did not show any expression.

These seven genes were expressed during the embryonic development of silkworm, and each of them may affect the embryonic development. Therefore, to verify the effects of the seven genes on the embryonic development of silkworm, RNAi was performed by synthesizing the siRNAs of the seven genes and injecting these siRNAs in diapause-free eggs laid by the Nistari. Given that the Fuyin*-lre* embryos stop developing at the late stage of gastrulation (about 48 h after egg laying), the microinjection was performed within 4 hours after egg laying. The RNAi results showed that among the 160 eggs injected with siRNAs of each gene, namely, BMSK0002759, KWMTBOMO02783, BMSK0002764, BMSK0002763, BMSK0002762, BMSK0002761, and BMSK0002760, 34, 66, 1, 78, 15, 4, and 77 embryos could develop to the late stage, respectively (Table 1). The injection of the siRNAs corresponding to BMSK0002764 and BMSK0002761 genes showed a considerable influence on the embryonic development. To verify the reliability of results, we performed a second injection for the BMSK0002764 and BMSK0002761 genes. Results showed that 3 of the 252 embryos injected with BMSK0002764 siRNAs and 19 of the 252 eggs injected with BMSK0002761 developed to the late stage (Table 1). Combining these RNAi results and considering each gene, we calculated the proportion of eggs that could develop to the late stage to the number of normal eggs. The proportion reached 1.28% for BMSK0002764 gene, 6.41% for the BMSK0002761 gene, and between 15.63% and 55.71% for the remaining five genes (Table 1). The RNAi results indicated that the BMSK0002764 gene plays an important role in the embryonic development of silkworm, followed by the BMSK0002761 gene. Given the chromosomal variation, nine genes, including BMSK0002764 and BMSK0002761, were silenced in Fuyin-*lre*. Such gene silencing resulted in embryonic lethality.

**Table 1:** RNAi results.

Gene	Total eggs	Number of dead eggs caused by injection	Number of normal eggs	Number of eggs developing to later stage	Number of eggs developing to later stage/ number of normal eggs (%)
EGFP	160	26	134	101	75.37
BMSK0002759	160	25	135	34	25.19
KWMTBOMO02783	160	16	144	66	45.83
BMSK0002764	412 (160+252)	100 (82+18)	312 (78+234)	4 (1+3)	1.28
BMSK0002763	160	20	140	78	55.71
BMSK0002762	160	64	96	15	15.63
BMSK0002761	412 (160+252)	53 (30+23)	359 (130+229)	23 (4+19)	6.41
BMSK0002760	160	15	145	77	53.10

Note: The first and second numbers in parentheses represent the number of eggs in the first and second injections, respectively.

**Fig 4:**
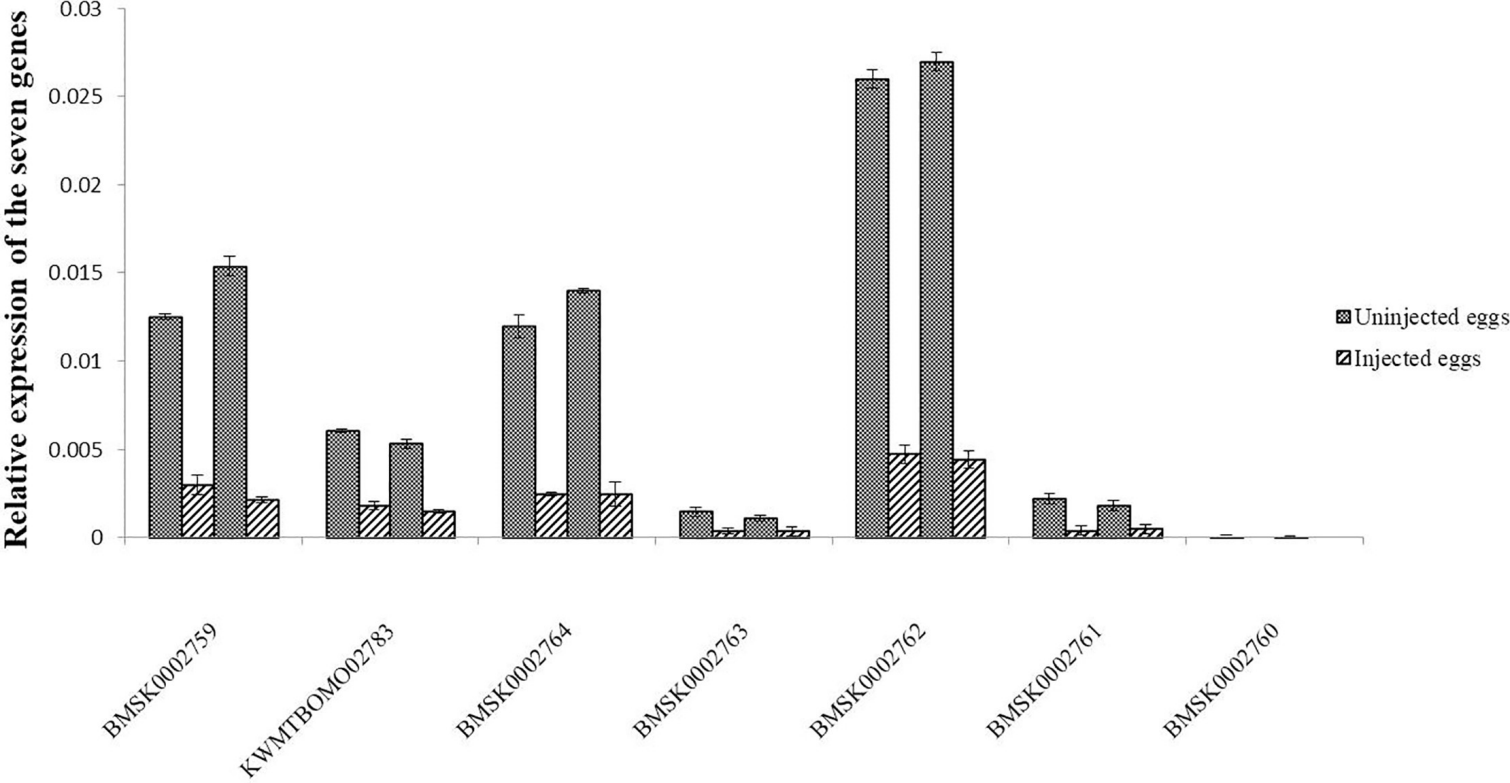
Relative expression levels of seven genes (BMSK0002759, KWMTBOMO02783, BMSK0002764, BMSK0002763, BMSK0002762, BMSK0002761, and BMSK0002760) in the eggs of Nistari at 24 and 48 h after RNAi. **The expression levels of BMSK0002759, KWMTBOMO02783, BMSK0002764, BMSK0002763, BMSK0002762, BMSK0002761, and BMSK0002760 were significantly decreased after RNAi.** The *BmActin3* was used as an internal control. Vertical bars represent the mean ± SE (n = 3).

To determine whether the siRNAs of each gene functioned after injection, we extracted the total RNA from 20 eggs 24 and 48 h after injection for qRT-PCR analysis. Results showed that except for the BMSK0002760 gene, the expression levels of the other six genes significantly decreased (Fig 4), indicating that the RNAi results were highly reliable. To verify whether injection of EGFP siRNAs will affect the expression of these 6 genes, we analyzed the expression of these 6 genes in eggs injected with ddH_2_O and EGFP siRNAs. The results showed that neither injection of water nor EGFP siRNAs would affect the expression of these six genes (S2 and S3 Figs).

BMSK0002764 is a gene similar to *semaphorin-1a* (*Sema-1a*) found in the silkworm genome database [31]. *Sema-1a* can promote the cellular growth of *Drosophila* through induction of key cell growth regulators [32] and prevent *Drosophila* olfactory projection neuron dendrites from mistargeting into select antennal lobe regions [33]. This gene is also required for optic lobe development in *A*. *aegypti* and highlights the behavioral importance of a functioning visual system in pre-adult mosquitoes [34]. Although semaphorin-1a has the above important functions and is involved in the neural development of *Drosophila* embryos [25], no report exists about the impact of *Sema-1a* on embryonic development. BMSK0002761 is a gene that is similar to RAS-GTPase-activating proteins (RASGAPs) found in the silkworm genome database [31]. RASGAPs are a group of tumor suppressors. They normally turn off the RAS pathway by catalyzing the RAS-GTP hydrolysis in tumorigenesis [35–37]. However, the effect of RASGAPs on the embryonic development has not been reported. The results of this study enrich the functions of *Sema-1a* and provide a theoretical basis for further research on the embryonic development of silkworm. Fuyin-*lre* is a lethal egg mutant. Therefore, it can provide genetic material for the development of genetic control technology of lepidopteran pests.

## Supporting information

S1 Table Primers used in iPCR, qRT-PCR and identification of 1102 bp sequence. (DOCX)

S2 Table siRNA sequences of candidate genes. (DOCX)

S1 Fig Relative expression levels of seven genes (BMSK0002759, KWMTBOMO02783, BMSK0002764, BMSK0002763, BMSK0002762, BMSK0002761, and BMSK0002760) in the eggs of Fuyin 24 and 48 h after egg laying. The *BmActin3* was used as an internal control. Vertical bars represent the mean ± SE (n = 3).
(TIF)

S2 Fig (TIF)

S3 Fig (TIF)

S1 File (PDF)

S2 File (XLSX)

S3 File (XLSX)

S4 File (XLS)

S1 Data (DOCX)
