# Supplementary material for: Semaphorin-1a-like gene plays an important role in the embryonic development of silkworm, Bombyx mori
Source: PLoS One. 2020 Oct 2;15(10):e0240193. doi: 10.1371/journal.pone.0240193 (PMC7531805; doi:10.1371/journal.pone.0240193)
Supplement: S1 Table — (DOCX) [file pone.0240193.s001.docx]

Table S1. Primers used in iPCR, qRT-PCR and identification of sequence 2

| Primer name | Forward primer | | Reverse primer |
| --- | --- | --- | --- |
| Downstream-iPCR1 | | TGTTGCTGGTGGTCATAGAT | AGAAAGGCACAGGATGCGAT |
| Downstream-iPCR2 | | CTCAAAGCTCTAAGGAAGCT | AGAAATGACCAGTACGTCTC |
| Upstream-iPCR1 | | TGACGTTACACAGCAATAGGT | GATCACATGCTTGACAATGGT |
| Upstream- iPCR 2 | | TTGGCTGTATGGTGCCGCCT | GGAAGTGTCTAAGGATGTAG |
| BMSK0002759-qRT-PCR | | AAGGAAGCCCTGCGAATCTA | ATAACCGTTGCCGTTTGAGT |
| KWMTBOMO02783-qRT-PCR | | GCACCAATAATGGCAACG | TGTGCAGCTATTTGTCTACT |
| BMSK0002764-qRT-PCR | | CGTCTTGTCTCGTTACCCT | ACGTTCGCTACCATATTGAC |
| BMSK0002763-qRT-PCR | | AGCAGCAACAAGGAACCAAG | TCTGAAAACCCATAATGAACTA |
| BMSK0002762-qRT-PCR | | AAGAAGATAGTGGTGCTGAA | CAATGGTAATGTCCTTGTGTA |
| BMSK0002761-qRT-PCR | | CACCTGCTGATGATATGGCAG | GCATATTGCTCCTGCTGTTC |
| BMSK0002760-qRT-PCR | | ATGGAGGGAGATTCCTGTCA | CCGAAACAATACTTAGGGTC |
| Sequence 1102 | | ACGACGTAATGGTACAGTCG | CATCGTGATGTAATGTGAGT |
