# Supplementary material for: Semaphorin-1a-like gene plays an important role in the embryonic development of silkworm, Bombyx mori
Source: PLoS One. 2020 Oct 2;15(10):e0240193. doi: 10.1371/journal.pone.0240193 (PMC7531805; doi:10.1371/journal.pone.0240193)
Supplement: S2 Table — (DOCX) [file pone.0240193.s002.docx]

Table S2. siRNA sequences of candidate genes

| Gene name | SiRNA sequence |
| --- | --- |
| BMSK0002764 | GCAGGAATACTGTCTACAA |
|  | GCGATCAATGGATGTCATT |
|  | GCTGTTAATGTTGCCTCTA |
| BMSK0002763 | CCAAGAGCCAATTCTGCAA |
|  | CCAACAGAATTTGACGAAA |
|  | GCTGCTGATTATAGTCTAA |
| BMSK0002762 | GGATTCCGACGAAGAAGAT |
|  | GGACTTGGTGATGAATCTA |
|  | GGAGTTCAAATGGAACCAA |
| BMSK0002761 | GCTATTTACCAGACGAATA |
|  | GGATGTAATCAACCAAGTT |
|  | CCAAGCTACGTAGGAACTT |
| BMSK0002760 | GCAGATGCATCAAGCATTT |
|  | GCTTCAGAGTTCCTAGTAA |
|  | GCCAAACGACTACAACGTA |
| BMSK0002759 | GGAACCTTCTAAGCAACAA |
|  | GCCTACAGAGAAGAGAGTT |
|  | GCAACGGTTATCTGGATTT |
| KWMTBOMO02783 | GGTCTGTAGCAAATTCTAA |
|  | GCAGGCGATAATTATGAAA |
|  | CCGGTTCCTGTCAATTCTT |

Nucleotide sequence of 1232 bp inserted sequence. The underlined sequence, which has a length of 130 bp, has multiple copies in the silkworm genome. The remaining sequence, which has a length of 1102 bp, has only a single copy on the silkworm genome, which is derived from 11413374 bp to 11414475 bp of chromosome 5.

CATCGTTCCCAGCGCTCCAGGCTTTTTTCACTCATATTCTACATCCTTAGACACTTCCAATAAGTTAGTATTTTAGAAATTCTCTAGAAATCTTATATATGGCAAGACACCGTTTGCCGGGTCAGCTAGTAAATTGCGTGGACAGAGATTTTCATCACCTGAAGAAGCTGTGGACGCCTACAAAGCGGCCATTTTGGAGACCCCAACTTCCGAATGGAATGGTTGCTTCAATGATTGGTTCCATCCTATGGAAAAATGTCTCAAATTTCGCGGAGAATACTTCGAAAACCAATAAATACATTTTTAAATAGTAATGTTGTGTGACTTCGTTAATTCCCGAAATTTTCAGTGCCGCCCTCGTACTATACTAATACACAAACACATGTTAATTTTCAAGTCTAGTTCATAACTGATTTAGTATATTATCAACAACACTCGAAGAGAATAAAGTTAATATTTTTATTTATTTTTTATTTATTTATTGCTTAGATGGGTGGACGAGCTCACTGCCCACCTGGTGTCAAGTGGTTACTGGAGCCCATAGACATTTACAACGTAAATGCGCCACCCACTTTGAGATATAAGTTCTAAGATCTCAGTATAGTTACAACGGCTGCCCTACCCTTCAAACCGAAACGCACTACTGCTTCACGGCAGAAATAGGCAGGGCGATGGTACTTACCCGTGCGGACTCACAAGAGGTCCTACCACCAGTAAAATAATCACATTATAGACATTTAACTAAAAAAATTTGGAATAATATTCCATTAAGGGTATAAAAATCATTAATCTTATTTTAATATTCATAAGAAATAAGTTAAATAATAAATTCAAGCACACATAATTGAAATAAATAGAACTATAGGCGCCGCCGTATTGGCCTTGGCTGCTCTGACGAGCGCCTTTCACTTTATCCCTACTCCGCGGCCGACCGTCAGCGACATGCAACACAGACGAAAAAGTTTGAGACAATGTAATAAAAGTTTCACTTTAAATGTAGTATAATATAATTACTGTTAGGTTTGCGTTTTATATCCTATAGATTTCTTTACGGGGATAAGTACGACGTAATGGTACAGTCGAAATAGATGAATATATTTTGGCTGTATGGTGCCGCCTTAAAATAGCATCCCCCGATCTTTTCCCGAGGTTGTCGCAAATGCAACTAAAGGATTCACCGAAAAATACAGAGCAACATTTTCCGAGAAGTATACCAGCGTACTGCGATTGCC

Nucleotide sequence of 1845 bp inserted sequence. The underlined sequence, which has a length of 186 bp, has multiple copies in the silkworm genome. The highlighted sequence is *bmmar1* transposon.

AAAACATCGGCACAAGTCTATCCAGATACCATTCTTGAGAAGGTAGTGAAGCTCCTTAACAACACCATGTTCAATAATCAAGAATGGTCCTTCCAGCAAGACTCGGCGCCAGGTCATAAAGCTCGGTCTACGCAGTCTTGGTTGGAAACGAACGTTTCGGACTTTATCAGAGCTGAAGACTGGCCGCCCGCCCGGTCCGGGGTAGGGCGCCGGCTGTCAGCGGCAGGAGTTTTTAGTGAGGTTCGACACCCACATACCCCACCTGCCGTGCGGGTGGAGATCAGGCGATTTTCTACAGTGAAAAAAAAAAAAAATCTTACTAACATACAAGTAAATACTTAGTCTGGCCATAAATACTGTTACAATTAAAATAAACAAAATATTACATTTGAATTTGGAATCTTTCATTTTTATATGATTGCTCATTGAGTTTTCTCATTTTGGCGCCAATACATTGTACAATATTTTGCGATAATAAAATGAAGTGGGGTGATAAAGAGAACCGAATCGCTGTGATTGCATTACACAAAGTAGGTATGGAGCCAAATACAATTTTTAAAACTCTCCATACGCTTGGTATTAGTAAAATGTTTGTGTACCGGGCTATTAATAGGTGCAATGAGACCTCCTCTGTTTGTGACAGAAAAAGATCTGGCCGTCCACGTAGTGTTCGTACGAAAAAGGTGGTCAAAGCAGTAAGGGAAAGAATTCGAAGAAATCCTGTCCGAAAGCAAAAGATTTTATCTCGGGAGATGAAGATAGCACCTAGAACCATGTCGCGTATTTTAAAAGATTACTTAGGACTTGCAGCCTATAAGAGATGTACTGGTCATTTCTTAACTGATAATTTAAAAGAGAATAGGGTGGTAAAATCGAAACAACTACTGAAGCGGTACGCAAAGGGAGGTCATAGAAAAATTTTGTTTACGGATGAGAATTTTTTTACAATTGAGCAACATTTTAACAAACAAAATGACTGTATTTATGCTCAAAGCTCTAAGGAAGCTTCCCAATTAGTCGACAGAGAGCAACATGGGCGCTATCCGACTTCAGTGATGGTTTGGTGGAGTATTAGCTATGATGGAGTGACTGGGCCATACTTTTGTGAAAAAGGTATCAAAACATCGGCACAAGTGAATCAAGATACCATTCTTGAGAAGGTAGTGAAGCCCCTTAACAACACCATGTTCAATAATCAAGAATGGTCCTTCCAGCAAGGCTCGGCGCCAGGTCATAAAGCTCGGTCTACGCAGTCTTGGTTGGAAACGAACGTTTCGGACTTCATCAGAGCTGAAGACTGGCCGTCGTCTAGTCCCGATCTTAATCCGCTGGATTATGATTTATGGTCAGTTTTAGAGAGTACGGCTTGCTCTAAACGCCATGATAATTTGGAGTCCCTAAAACAATCCGTACGATTGGCAGTGAAAATTTTTCCCATGGAAAGAGTGCGTGCTTCTATTGATAACTGGCCTCAACGTTTAAAGGACTGTATTGCAGCCAATGGAGACCACTTCGAATAAGCTTTTTATACTTTAAATTGTTTTATATTTATGTATTAAACTAACACACTGTAAAAGTAATAAATGTTATTTGCCATAGATTTTTTTTTGTTTTTCTTTGTAACAGTATTTATGGCCAGACTAAGTATACAACTAATTATGGTCTCATTATTGTAAACTCAAACTGGTTCACAAACGCTGTCCCCTTCTGTGGCAAGGCTACAATAAATTGATACAATAAATATTTACGATTTTGTCATCGTAATTACTTTGAGTTCAAGCGGTATTCTCATCAGAATAAGCAATTACA1TAAATTCTCAGTGTTAAGTGGTTACCGAAGACATT
