# Supplementary figures and images for: Semaphorin-1a-like gene plays an important role in the embryonic development of silkworm, Bombyx mori
Source: PLoS One. 2020 Oct 2;15(10):e0240193. doi: 10.1371/journal.pone.0240193 (PMC7531805; doi:10.1371/journal.pone.0240193)

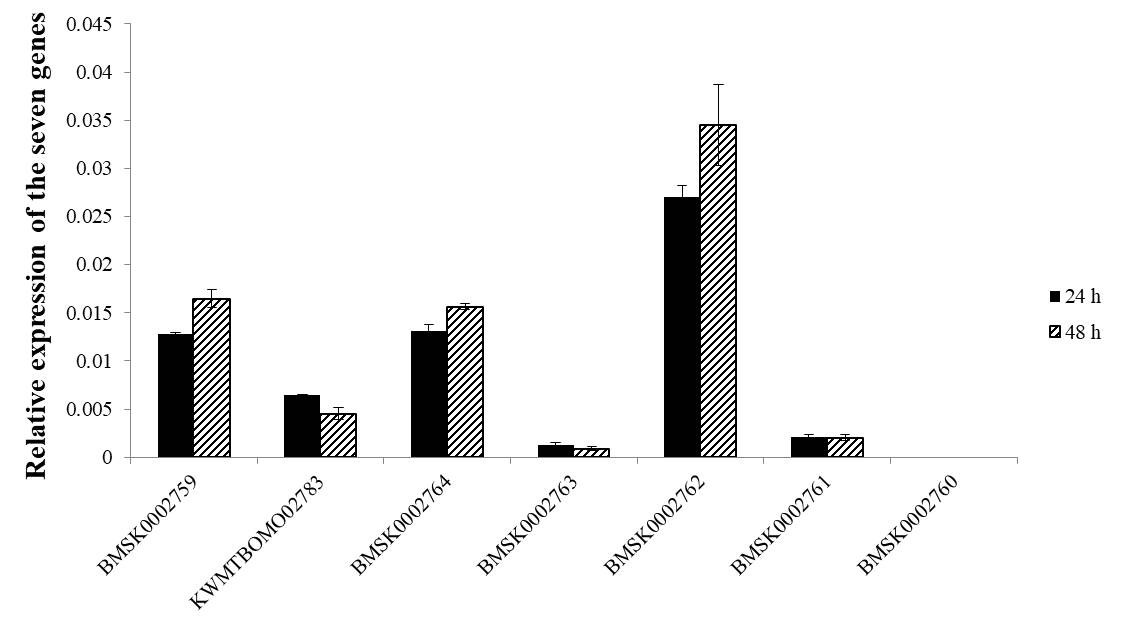

Supplement: S1 Fig — The BmActin3 was used as an internal control. Vertical bars represent the mean ± SE (n = 3). (TIF) [file pone.0240193.s003.tif]

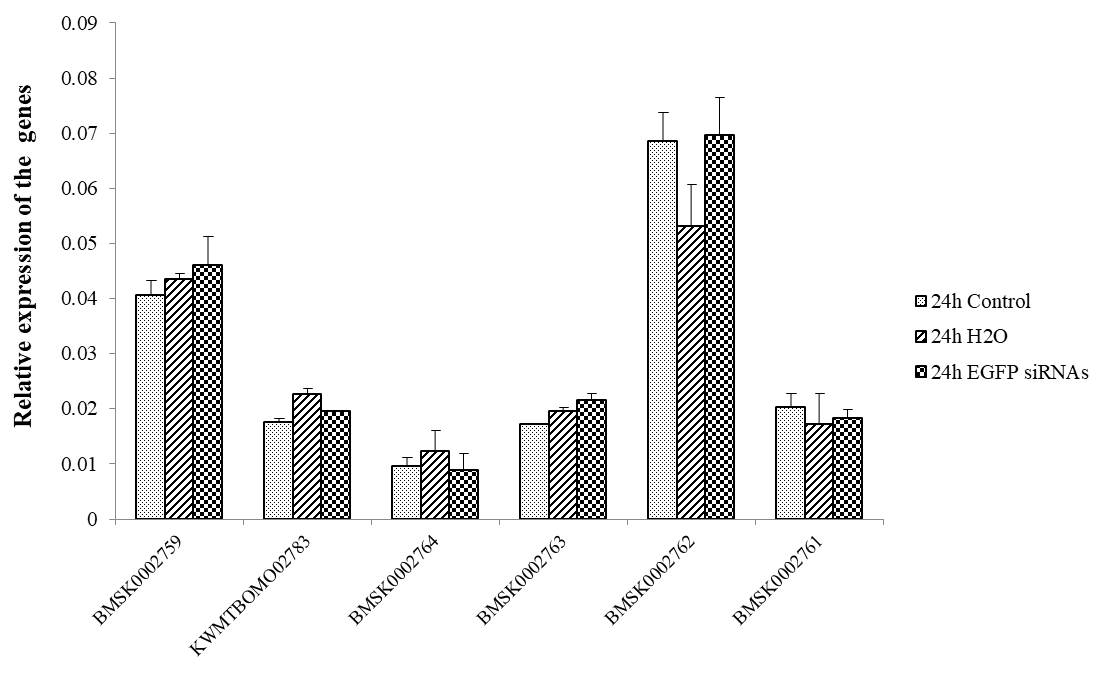

Supplement: S2 Fig — (TIF) [file pone.0240193.s004.tif]

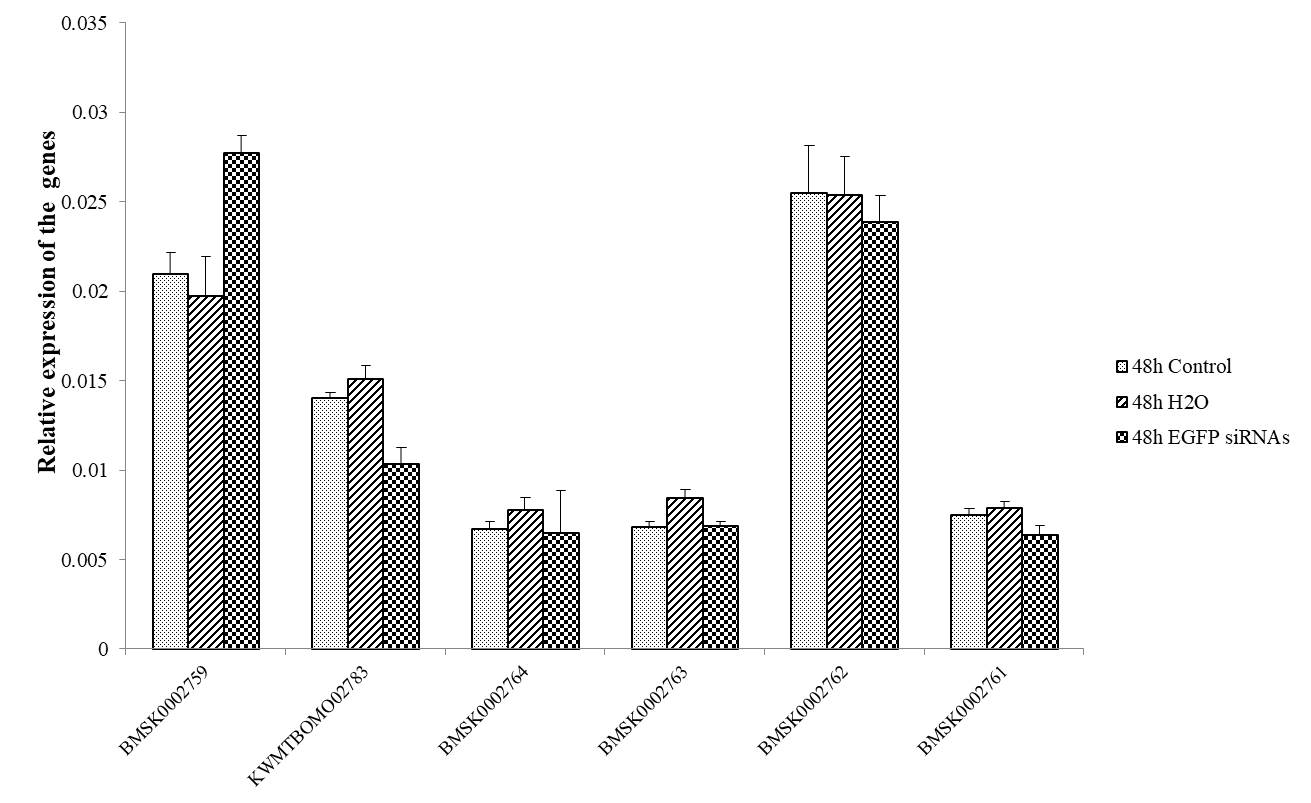

Supplement: S3 Fig — (TIF) [file pone.0240193.s005.tif]

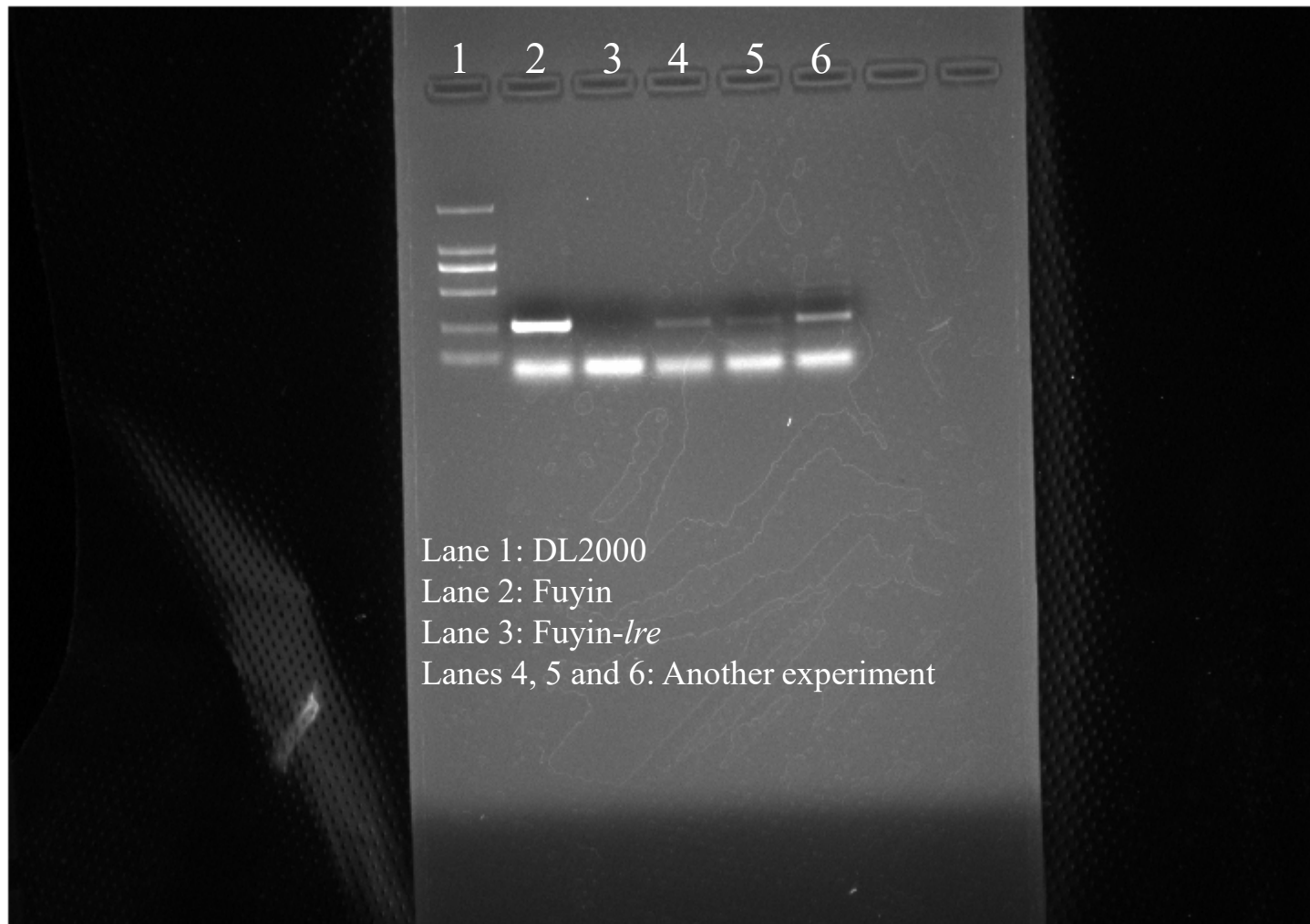

Supplement: S1 File — (PDF) [file pone.0240193.s006.pdf]
