# Supplementary material for: Semaphorin-1a-like gene plays an important role in the embryonic development of silkworm, Bombyx mori
Source: PLoS One. 2020 Oct 2;15(10):e0240193. doi: 10.1371/journal.pone.0240193 (PMC7531805; doi:10.1371/journal.pone.0240193)
Supplement: S1 Data — (DOCX) [file pone.0240193.s010.docx]

CATCGTTCCCAGCGCTCCAGGCTTTTTTCACTCATATTCTACATCCTTAGACACTTCCAATAAGTTAGTATTTTAGAAATTCTCTAGAAATCTTATATATGGCAAGACACCGTTTGCCGGGTCAGCTAGTAAATTGCGTGGACAGAGATTTTCATCACCTGAAGAAGCTGTGGACGCCTACAAAGCGGCCATTTTGGAGACCCCAACTTCCGAATGGAATGGTTGCTTCAATGATTGGTTCCATCCTATGGAAAAATGTCTCAAATTTCGCGGAGAATACTTCGAAAACCAATAAATACATTTTTAAATAGTAATGTTGTGTGACTTCGTTAATTCCCGAAATTTTCAGTGCCGCCCTCGTACTATACTAATACACAAACACATGTTAATTTTCAAGTCTAGTTCATAACTGATTTAGTATATTATCAACAACACTCGAAGAGAATAAAGTTAATATTTTTATTTATTTTTTATTTATTTATTGCTTAGATGGGTGGACGAGCTCACTGCCCACCTGGTGTCAAGTGGTTACTGGAGCCCATAGACATTTACAACGTAAATGCGCCACCCACTTTGAGATATAAGTTCTAAGATCTCAGTATAGTTACAACGGCTGCCCTACCCTTCAAACCGAAACGCACTACTGCTTCACGGCAGAAATAGGCAGGGCGATGGTACTTACCCGTGCGGACTCACAAGAGGTCCTACCACCAGTAAAATAATCACATTATAGACATTTAACTAAAAAAATTTGGAATAATATTCCATTAAGGGTATAAAAATCATTAATCTTATTTTAATATTCATAAGAAATAAGTTAAATAATAAATTCAAGCACACATAATTGAAATAAATAGAACTATAGGCGCCGCCGTATTGGCCTTGGCTGCTCTGACGAGCGCCTTTCACTTTATCCCTACTCCGCGGCCGACCGTCAGCGACATGCAACACAGACGAAAAAGTTTGAGACAATGTAATAAAAGTTTCACTTTAAATGTAGTATAATATAATTACTGTTAGGTTTGCGTTTTATATCCTATAGATTTCTTTACGGGGATAAGTACGACGTAATGGTACAGTCGAAATAGATGAATATATTTTGGCTGTATGGTGCCGCCTTAAAATAGCATCCCCCGATCTTTTCCCGAGGTTGTCGCAAATGCAACTAAAGGATTCACCGAAAAATACAGAGCAACATTTTCCGAGAAGTATACCAGCGTACTGCGATTGCC

Nucleotide sequence of 1845 bp inserted sequence. The underlined sequence, which has a length of 186 bp, has multiple copies in the silkworm genome. The highlighted sequence is *bmmar1* transposon.

AAAACATCGGCACAAGTCTATCCAGATACCATTCTTGAGAAGGTAGTGAAGCTCCTTAACAACACCATGTTCAATAATCAAGAATGGTCCTTCCAGCAAGACTCGGCGCCAGGTCATAAAGCTCGGTCTACGCAGTCTTGGTTGGAAACGAACGTTTCGGACTTTATCAGAGCTGAAGACTGGCCGCCCGCCCGGTCCGGGGTAGGGCGCCGGCTGTCAGCGGCAGGAGTTTTTAGTGAGGTTCGACACCCACATACCCCACCTGCCGTGCGGGTGGAGATCAGGCGATTTTCTACAGTGAAAAAAAAAAAAAATCTTACTAACATACAAGTAAATACTTAGTCTGGCCATAAATACTGTTACAATTAAAATAAACAAAATATTACATTTGAATTTGGAATCTTTCATTTTTATATGATTGCTCATTGAGTTTTCTCATTTTGGCGCCAATACATTGTACAATATTTTGCGATAATAAAATGAAGTGGGGTGATAAAGAGAACCGAATCGCTGTGATTGCATTACACAAAGTAGGTATGGAGCCAAATACAATTTTTAAAACTCTCCATACGCTTGGTATTAGTAAAATGTTTGTGTACCGGGCTATTAATAGGTGCAATGAGACCTCCTCTGTTTGTGACAGAAAAAGATCTGGCCGTCCACGTAGTGTTCGTACGAAAAAGGTGGTCAAAGCAGTAAGGGAAAGAATTCGAAGAAATCCTGTCCGAAAGCAAAAGATTTTATCTCGGGAGATGAAGATAGCACCTAGAACCATGTCGCGTATTTTAAAAGATTACTTAGGACTTGCAGCCTATAAGAGATGTACTGGTCATTTCTTAACTGATAATTTAAAAGAGAATAGGGTGGTAAAATCGAAACAACTACTGAAGCGGTACGCAAAGGGAGGTCATAGAAAAATTTTGTTTACGGATGAGAATTTTTTTACAATTGAGCAACATTTTAACAAACAAAATGACTGTATTTATGCTCAAAGCTCTAAGGAAGCTTCCCAATTAGTCGACAGAGAGCAACATGGGCGCTATCCGACTTCAGTGATGGTTTGGTGGAGTATTAGCTATGATGGAGTGACTGGGCCATACTTTTGTGAAAAAGGTATCAAAACATCGGCACAAGTGAATCAAGATACCATTCTTGAGAAGGTAGTGAAGCCCCTTAACAACACCATGTTCAATAATCAAGAATGGTCCTTCCAGCAAGGCTCGGCGCCAGGTCATAAAGCTCGGTCTACGCAGTCTTGGTTGGAAACGAACGTTTCGGACTTCATCAGAGCTGAAGACTGGCCGTCGTCTAGTCCCGATCTTAATCCGCTGGATTATGATTTATGGTCAGTTTTAGAGAGTACGGCTTGCTCTAAACGCCATGATAATTTGGAGTCCCTAAAACAATCCGTACGATTGGCAGTGAAAATTTTTCCCATGGAAAGAGTGCGTGCTTCTATTGATAACTGGCCTCAACGTTTAAAGGACTGTATTGCAGCCAATGGAGACCACTTCGAATAAGCTTTTTATACTTTAAATTGTTTTATATTTATGTATTAAACTAACACACTGTAAAAGTAATAAATGTTATTTGCCATAGATTTTTTTTTGTTTTTCTTTGTAACAGTATTTATGGCCAGACTAAGTATACAACTAATTATGGTCTCATTATTGTAAACTCAAACTGGTTCACAAACGCTGTCCCCTTCTGTGGCAAGGCTACAATAAATTGATACAATAAATATTTACGATTTTGTCATCGTAATTACTTTGAGTTCAAGCGGTATTCTCATCAGAATAAGCAATTACA1TAAATTCTCAGTGTTAAGTGGTTACCGAAGACATT
